# Supplementary material for: Lightweight error-tolerant edge detection using memristor-enabled stochastic computing
Source: Nat Commun. 2025 May 16;16:4550. doi: 10.1038/s41467-025-59872-2 (PMC12084603; doi:10.1038/s41467-025-59872-2)
Supplement: Supplementary file 1 — Supplementary Information [file 41467_2025_59872_MOESM1_ESM.pdf]

Supplementary Information for
Lightweight error-tolerant edge detection using memristor-enabled stochastic computing

Author list
Lekai Song[1], Pengyu Liu[1], Jingfang Pei[1], Yang Liu[1,2], Songwei Liu[1], Shengbo Wang[3], Leonard W. T. Ng[4], Tawfique Hasan[5], Kong-Pang Pun[1], Shuo Gao[3], Guohua Hu[1,*]

Affiliations
[1]Department of Electronic Engineering, The Chinese University of Hong Kong, Shatin, N. T., Hong Kong S. A. R., China
[2]Shun Hing Institute of Advanced Engineering, The Chinese University of Hong Kong, Shatin, N. T., Hong Kong S. A. R., China
[3]School of Instrumentation and Optoelectronic Engineering, Beihang University, Beijing 100191, China
[4]School of Materials Science and Engineering, Nanyang Technological University, Singapore 639798, Singapore
[5]Cambridge Graphene Centre, University of Cambridge, Cambridge CB3 0FA, UK

*Correspondence to: ghhu@ee.cuhk.edu.hk

This file contains:
Supplementary Figures 1-12
Supplementary Tables 1-3
Supplementary References

Supplementary Figures

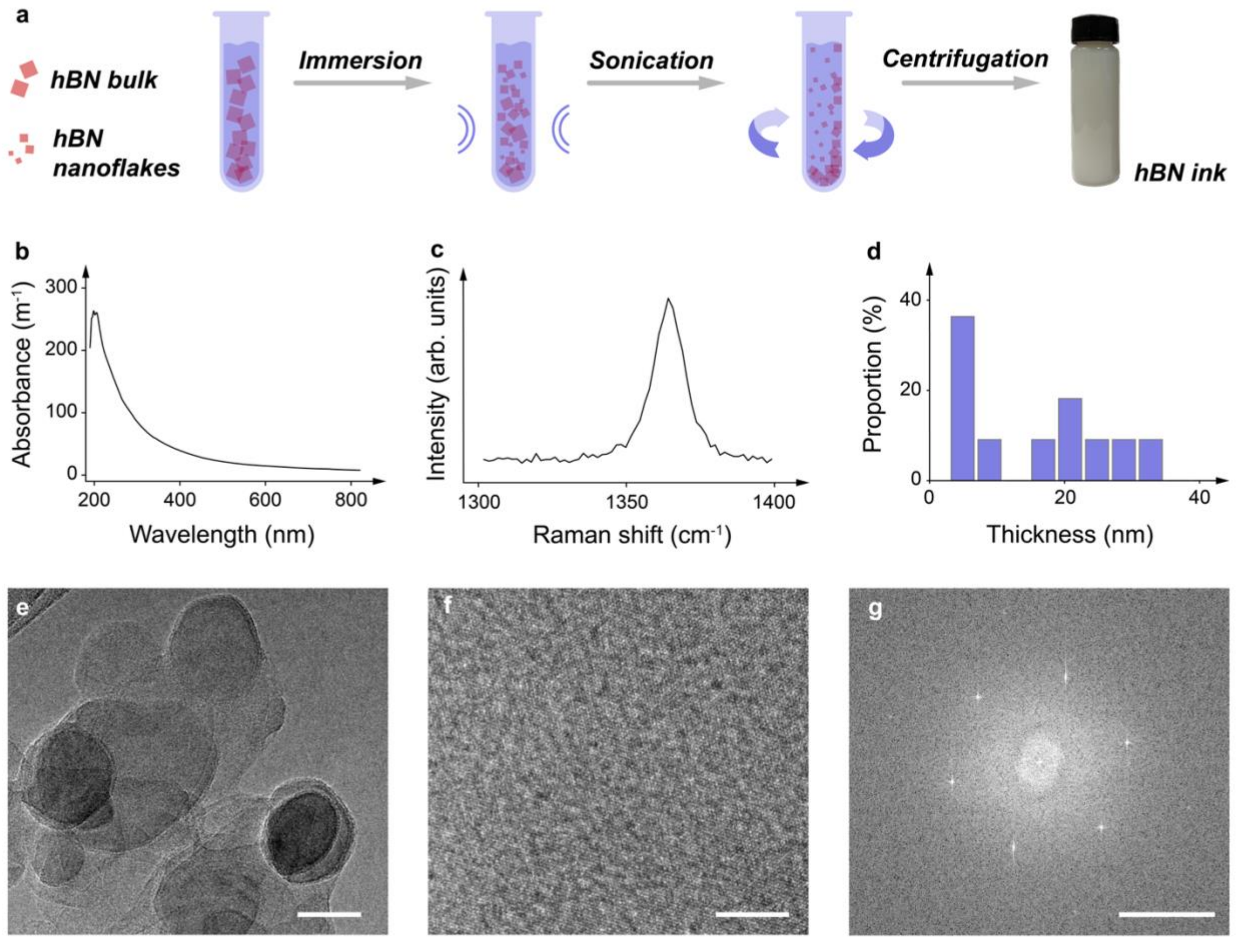

Supplementary Figure 1. hBN ink preparation and characterizations. (a) Schematic liquid-phase exfoliation of hBN, following our previous report Ref. (*1*). To specify, hBN powder (10 g L^{-1}) and polyvinylpyrrolidone (1 g L^{-1}) are mixed into isopropanol in a sonication tube in ambient condition. The mixture undergoes 48-hour bath sonication at ~10 °C to facilitate exfoliation and dispersion. Subsequently, the dispersion of the as-exfoliated hBN nanoflakes in isopropanol is centrifuged at 4,000 rpm for 30 minutes to remove insufficiently exfoliated aggregates. The supernatant is then carefully decanted and collected. Controlled volumes of isopropanol and 2-butanol are added to formulate a stable hBN ink in isopropanol/2-butanol (90/10 vol%), with a concentration of ~1 g L^{-1}. (b) Absorption spectrum of a typical hBN ink (diluted to 10%), showing no featureless absorption peaks. (c) Raman spectrum of the as-exfoliated exfoliated hBN nanoflakes. (d) Histogram of the thickness of the as-exfoliated hBN nanoflakes characterized by atomic force microscopy (AFM). The averaged thickness is ~9.31 nm, excluding the thick (>20 nm) aggregates formed during the AFM sample preparation process. (e, f) Transmission electron microscopic (TEM) images of the as-exfoliated hBN nanoflakes, and (g) the corresponding fast Fourier transform pattern, confirming the hexagonal lattice structure of as-exfoliated hBN nanoflakes. Scale bars – (e) 50 nm, (f) 5 nm, and (g) 5 nm^{-1}.

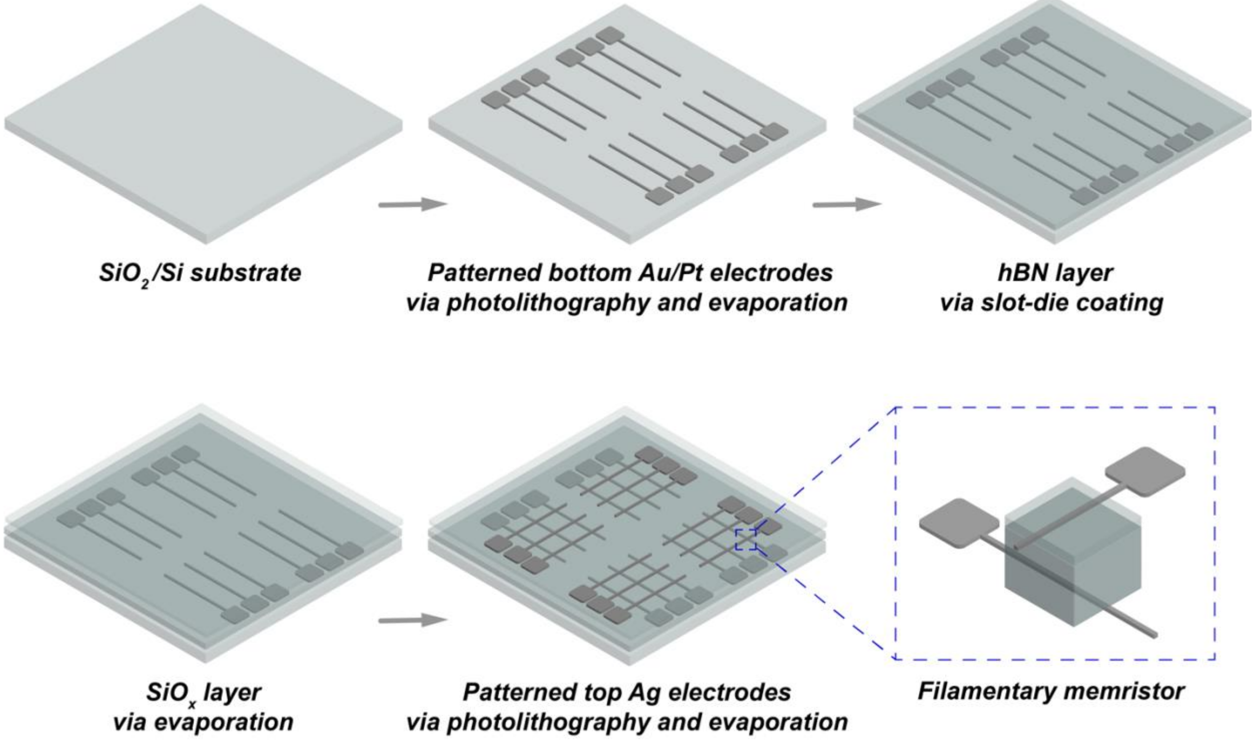

Supplementary Figure 2. Memristor fabrication. Starting from a cleansed SiO_2/Si substrate, the bottom Au/Pt electrodes are patterned using photolithography and evaporation. A hBN layer is deposited on the bottom electrodes using slot-die coating. A SiO_x layer is then deposited using evaporation to minimize the wash-off of hBN during the subsequent patterning processes and increase the fabrication yield to 100%, while preserving the switching behavior of the filamentary memristors. Next, the top silver electrodes are patterned using photolithography and evaporation. As such, the memristor devices are developed at the cross-points of the top and bottom electrodes, in a vertical Au/Pt/hBN/SiO_x/Ag configuration.

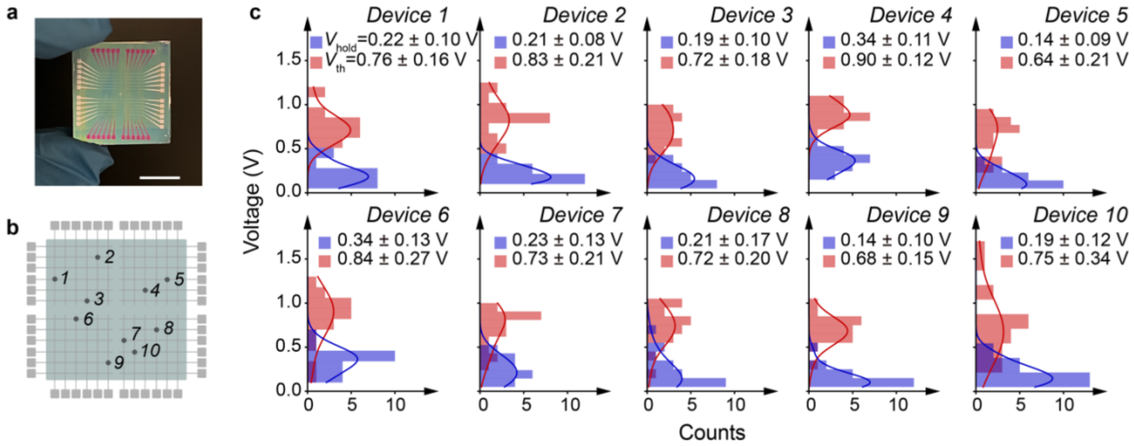

Supplementary Figure 3. Device-to-device stochasticity test. (a) 12×12 memristor array in a crossbar configuration, with a fabrication yield approximating 100% (replotted from Fig. 2b), and (b) the corresponding schematic array showing the devices randomly selected for the sampling test. (c) Distributions of the measured V_{hold} and V_{th} of sampled devices for 20 sweeping cycles, along with the corresponding Gaussian fittings, indicating minimal device-to-device stochasticity – V_{hold} variation 6.6%, V_{th} variation 7.4%. The device-to-device variations are defined using the standard deviations of the mean V_{hold} (V_{th}) values.

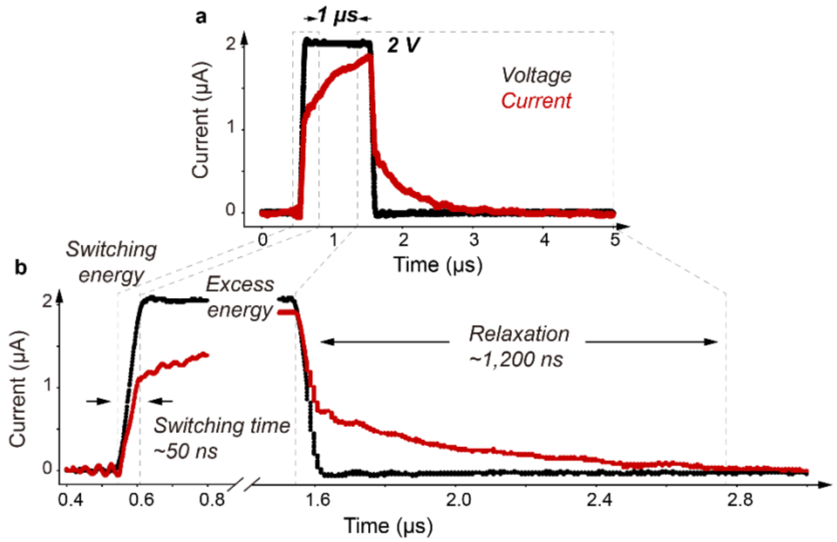

Supplementary Figure 4. Switching speed and energy consumption. (a) Electrical response to 1 μs pulsed signal, showing (b) a switching time of ~50 ns, a switching energy consumption of ~33 fJ, and a relaxation time of ~1,200 ns.

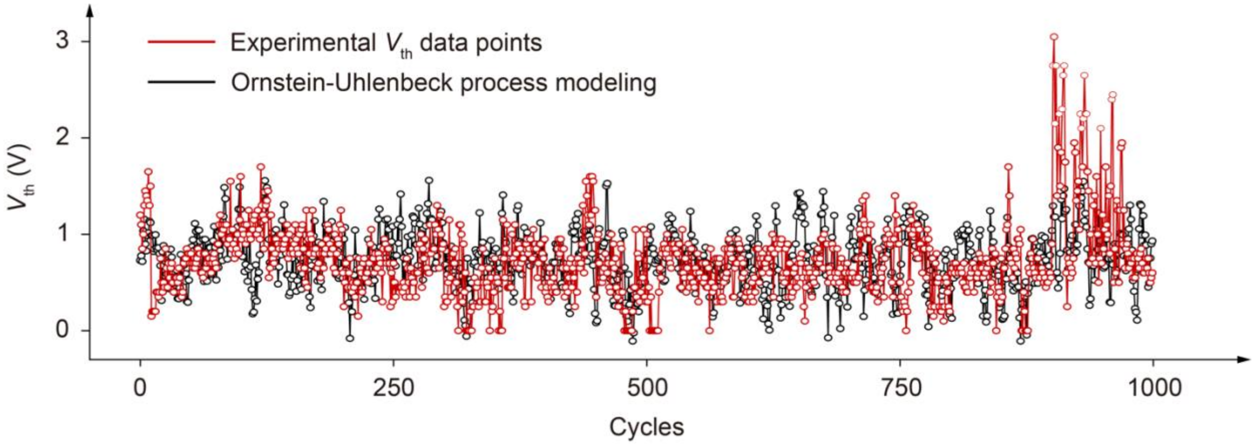

Supplementary Figure 5. Stability test of the memristor switching stochasticity. Ornstein-Uhlenbeck process modeling on the measured threshold voltage V_{th} of the hBN filamentary memristor across the 1,000 consecutive sweeping cycles present in Fig. 2d. The experimental V_{th} data points well fit those from the Ornstein-Uhlenbeck process modelling, where $dV_{\mathrm{th,t}} = \theta(\mu - V_{\mathrm{th,t}}) + \sigma dW_{\mathrm{t}} = 0.306 \times (0.729 - V_{\mathrm{th,t}}) + 0.284 \times dW_{\mathrm{t}}$. Ornstein-Uhlenbeck process describes a stochastic process in a dynamical system (*2*). A dW_{t} denotes the variation of a Wiener process, i.e. a real-valued continuous-time stochastic process. This proves the stability of the switching stochasticity of our memristors in prolonged switching operations. At around 900-th cycle, the device is momentarily stuck at the high resistance states and thus hard to switch on probably due to the ambient disturbances but then returns to normal.

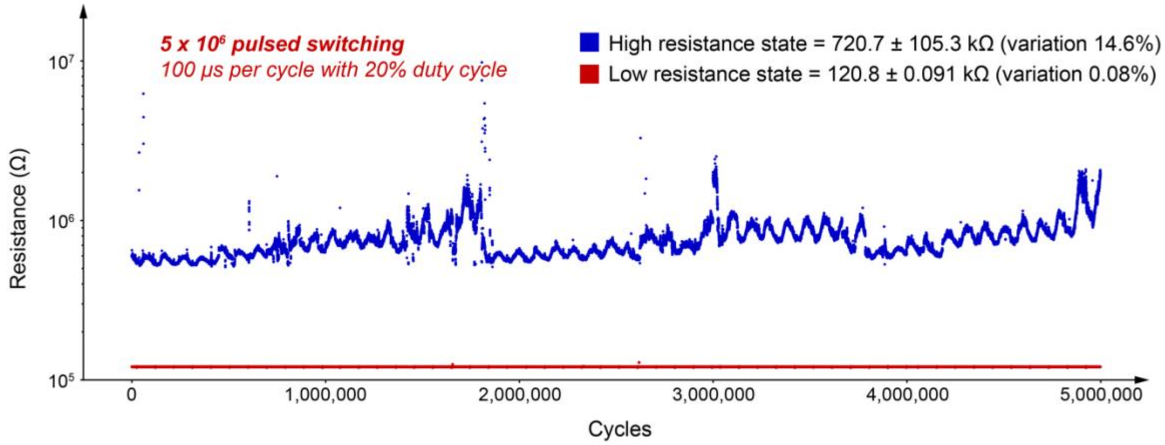

Supplementary Figure 6. Endurance test. Endurance test of a typical memristor undergoing 5×10^6 consecutive test cycles under pulsed stimuli. For each test cycle, a 20 µs voltage pulse of 10 V is set to fully program the memristor and an 80 µs voltage pulse of 0.1 V is set to read the output. The output of the memristor is amplified by an operational amplifier and measured by an oscilloscope. The high (i.e. off) and low (i.e. on) resistance states in each test cycle are computed based on the oscilloscope measurement, and plotted in blue and red, respectively. Both the states remain stable throughout the full test.

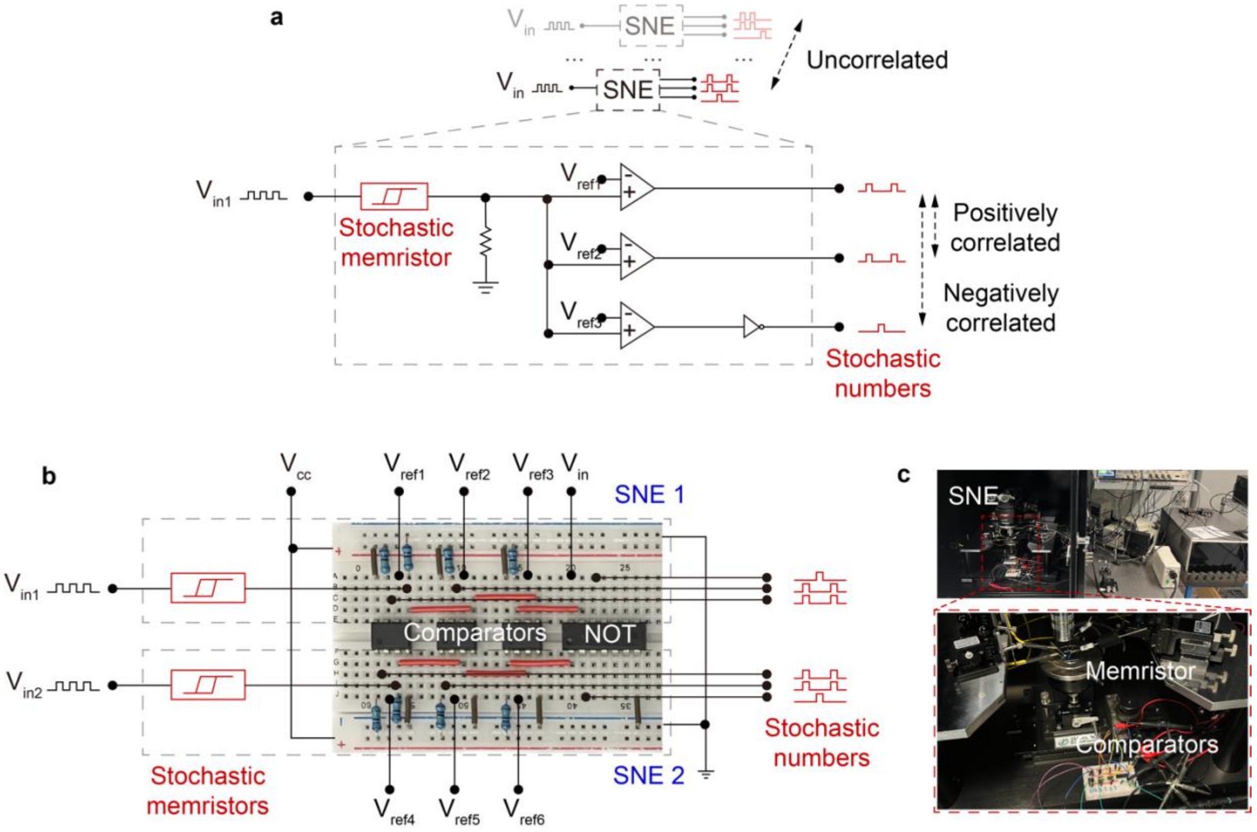

Supplementary Figure 7. The circuit design and experimental hardware realization of the SNEs. (a) Circuit design (replotted from Fig. 2a) and (b) hardware realization of SNEs. (c) Experimental setup. To build the SNEs, the memristors are tested on a probe station and connected to the logic gates and other electronic devices on a breadboard. The electronic components for the SNE realization include comparators, NOT gates, and resistors. Note that the voltage supply of the NOT gates is synchronized with V_{in} to the memristors to avoid output during the pulse intervals.

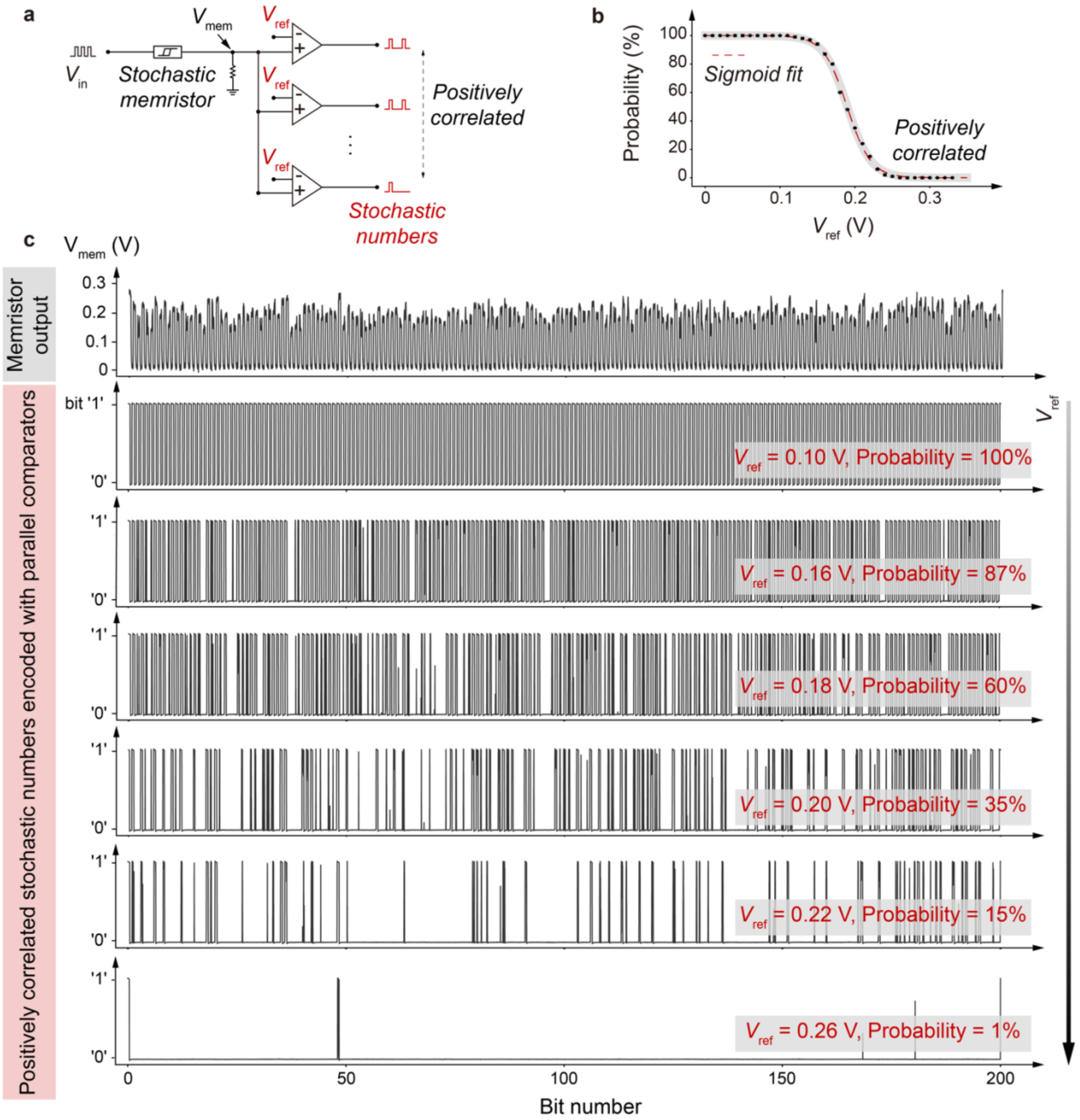

Supplementary Figure 8. Example showing the encoding of positive correlated stochastic numbers. (a) Schematic SNE in positive correlation, replotted from Fig. 2a, to encode the positively correlated stochastic numbers. (b) $P_{positive}$ - V_{ref} relation of the SNE in positive correlation, replotted from Fig. 2g, and (c) the corresponding experimental results. For a fixed memristor voltage output V_{mem} (the first row), the SNE configured with two parallel comparators encodes positively correlated stochastic numbers. The probability of the encoded stochastic numbers, i.e. the probability of 1s in the stochastic numbers, decreases as V_{ref} increases, aligned well with the sigmoid fit in (b).

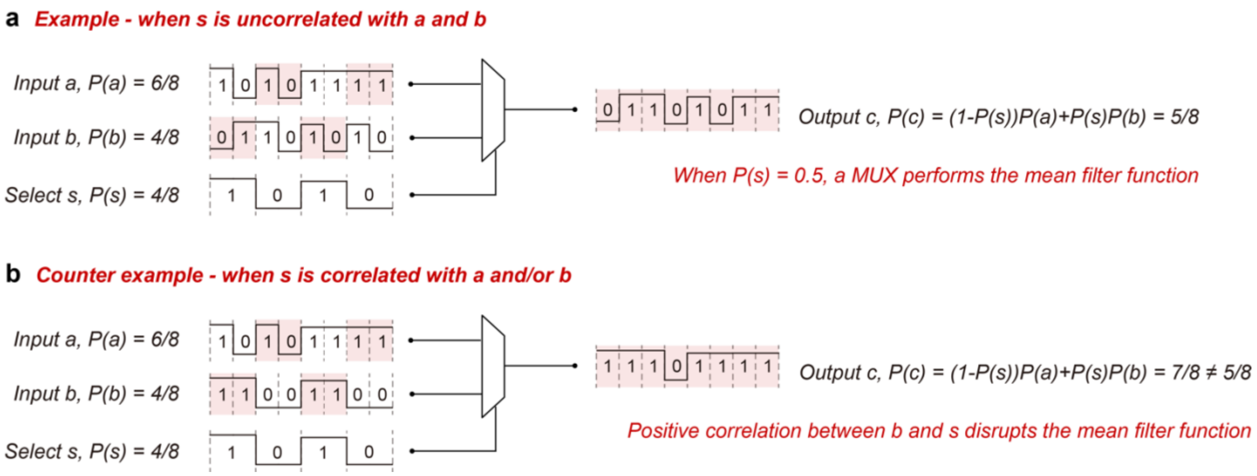

Supplementary Figure 9. Stochastic MUX logic. (a) An example showing the working principle of the stochastic MUX logic as a mean filter. Given that stochastic MUX logic follows $P(c) = (1 - P(s))P(a) + P(s)P(b)$ as summarized in Supplementary Table 2, a MUX can perform the mean filter function when $P(s) = 0.5$, i.e. $P(c) = 0.5(P(a) + P(b))$. Note that in this case the select s should be uncorrelated with the inputs a and b, and that the frequency of s is half of that of the inputs to ensure that both the inputs participate in the logic operations. For example, as shown, s selects bits from two input channels according to the bit level of s, where the bits as marked in blue are selected. As such, the MUX outputs the mean value (5/8) of inputs (6/8 and 4/8). (b) A counter example. A MUX no longer performs the mean filter function when the select s is correlated with the inputs a and/or b. As shown, a strong positive correlation between b and s disrupts the mean filter function, because s completely accepts b as part of the output c, instead of selecting bits from b with a probability.

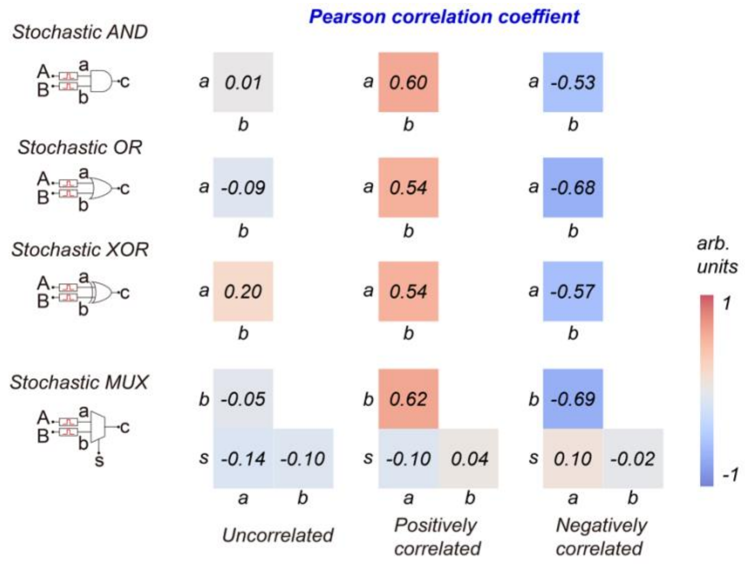

Supplementary Figure 10. Pearson correlation coefficients of the stochastic logics. Pairwise Pearson correlation of the inputs a, b, and s (if MUX) of the stochastic logics in the uncorrelation and correlation conditions. The Pearson correlation coefficient is defined as $\rho(a,b) = \frac{wz-xy}{\sqrt{(w+x)(w+y)(x+z)(y+z)}}$, where w, x, y, and z represent the counts of 1-1, 1-0, 0-1, and 0-0 pairs for the two stochastic numbers a and b, respectively.

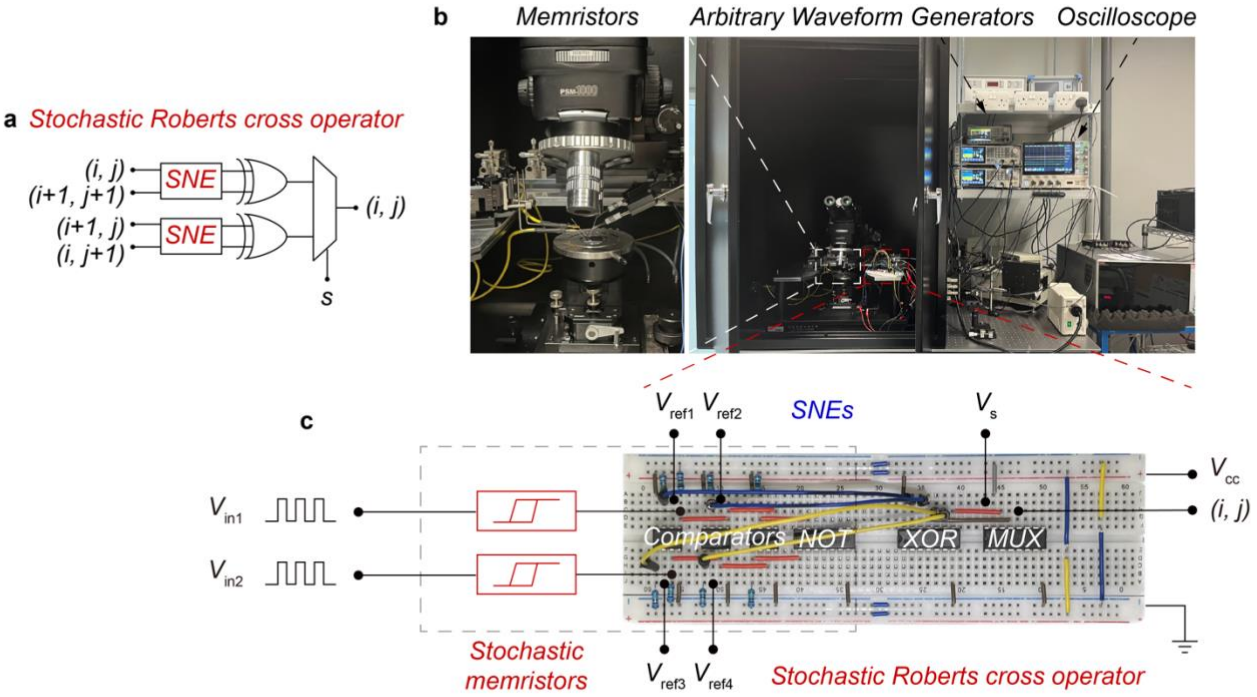

Supplementary Figure 11. The circuit design and experimental hardware realization of the stochastic Roberts cross operator. (a) Circuit design (replotted from Fig. 4b). (b) Experimental setup. (c) Hardware realization. To build the stochastic Roberts cross operator, the memristors are tested on a probe station and connected to the logic gates and other electronic devices on a breadboard. The electronic components for the SNE realization include comparators, NOT gates, and resistors. The reference voltages of the comparators V_{ref}, the voltage supply V_{cc}, and the pulsed voltage signals V_{in} are powered by the arbitrary waveform generators. The output of the stochastic Roberts cross operator (i, j) is measured by the oscilloscope. Note that the frequency of the select V_{s} of the MUX is half of that of V_{in} to ensure that both the inputs participate in the logical operations.

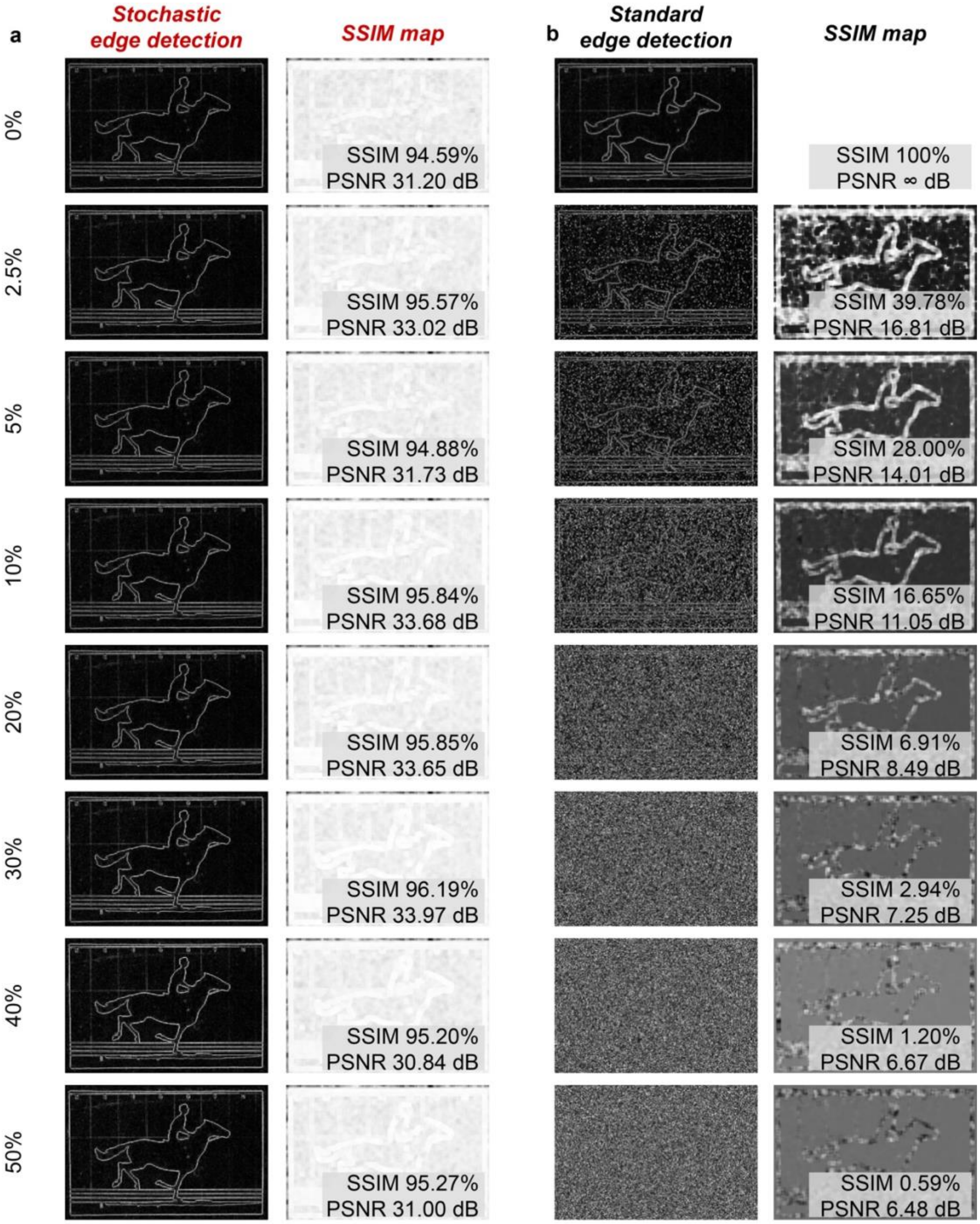

Supplementary Figure 12. Error-tolerance test. (a) Stochastic and (b) standard edge detection results and the corresponding SSIM maps with bit-flip injection at a ratio of 0%, 2.5%, 5%, 10%, 20%, 30%, 40%, and 50%. For the stochastic edge detection, the high SSIM (>90%) and PSNR (>30 dB) prove that the bit-flip injection does not degrade the edge detection performance. In contrast, a low level of bit-flip injection significantly degrades the performance of the standard algorithmic edge detection.

Supplementary Table 1. Comparison between the stochastic number encoders implemented using linear feedback shift registers (LFSR), magnetic tunnel junction (MTJ), Mott memristors, filamentary memristors, and our memristors.

	Max encoding speed (kbit s^{-1})	Endurance (cycles)	Transistor/ memristor count
LFSR (*3*)	10^7	/	1194
LFSR (*4*)	10^5	/	/
MTJ (*3*)	10^4	/	4
Mott (*5*)	400	$>1.05\times10^5$	/
Mott (*6*)	263~3,846	/	/
Mott (*7*)	/	5×10^4	3
Filamentary (*8*)	1	10^6	3
**This work**	**455**	$\mathbf{>5\times10^6}$	**3**

Supplementary Table 2. Statistical formulas of the stochastic logics. Stochastic logic operations with AND, OR, XOR, and MUX in the uncorrelation, positive correlation, and negative correlation configurations are presented. The stochastic numbers are assumed in a unipolar format *(9)*.

	Uncorrelated	Positively correlated	Negatively correlated
AND	$P(c) = P(a)P(b)$	$P(c) = \text{Min}(P(a), P(b))$	$P(c) = \text{Max}(P(a) + P(b) - 1,0)$
OR	$P(c) = P(a) + P(b) - P(a)P(b)$	$P(c) = \text{Max}(P(a), P(b))$	$P(c) = \text{Min}(1, P(a) + P(b))$
XOR	$P(c) = P(a) + P(b) - 2P(a)P(b)$	$P(c) = \lvert P(a) - P(b)\rvert$	$P(c) = P(a) + P(b)$, if $P(a) + P(b) \leq 1$; $P(c) = 2 - \big(P(a) + P(b)\big)$, otherwise.
MUX	$P(c) = \big(1 - P(s)\big)P(a) + P(s)P(b)$, if s is uncorrelated with a and b		

Supplementary Table 3. Power consumption of logic chips. The data are from the product datasheets.

	Chip	Power (mW)	Channel	Power/channel (mW)
AND	SN74HC08N	120.0	4	30.0
OR	HD74LS32P	25.7	4	6.4
XOR	HD74LS86P	32.0	4	8.0
MUX	SN74LS157N	96.0	4	24.0
Comparator	LM393	14.4	2	7.2

Supplementary References

1. L. Song, P. Liu, J. Pei, F. Bai, Y. Liu, S. Liu, Y. Wen, L. W. T. Ng, K. P. Pun, S. Gao, M. Q. H. Meng, T. Hasan, G. Hu, Spiking neurons with neural dynamics implemented using stochastic memristors. *Advanced Electronic Materials* **2300564**, 1–9 (2023).

2. S. Dutta, G. Detorakis, A. Khanna, B. Grisafe, E. Neftci, S. Datta, Neural sampling machine with stochastic synapse allows brain-like learning and inference. *Nature Communications* **13**, 2571 (2022).

3. W. A. Borders, A. Z. Pervaiz, S. Fukami, K. Y. Camsari, H. Ohno, S. Datta, Integer factorization using stochastic magnetic tunnel junctions. *Nature* **573**, 390–393 (2019).

4. P. Knag, W. Lu, Z. Zhang, A native stochastic computing architecture enabled by memristors. *IEEE Transactions on Nanotechnology* **13**, 283–293 (2014).

5. Y. Seo, Y. Park, P. Hur, M. Jo, J. Heo, B. J. Choi, J. Son, Promotion of Probabilistic Bit Generation in Mott Devices by Embedded Metal Nanoparticles. *Advanced Materials* **2402490** (2024).

6. H. Rhee, G. Kim, H. Song, W. Park, D. H. Kim, J. H. In, Y. Lee, K. M. Kim, Probabilistic computing with NbOx metal-insulator transition-based self-oscillatory pbit. *Nature Communications* **14**, 1–8 (2023).

7. S. Deng, T. J. Park, H. Yu, A. Saha, A. N. M. N. Islam, Q. Wang, A. Sengupta, S. Ramanathan, Hydrogenated VO2 Bits for Probabilistic Computing. *IEEE Electron Device Letters* **44**, 1776–1779 (2023).

8. K. S. Woo, J. Kim, J. Han, W. Kim, Y. H. Jang, C. S. Hwang, Probabilistic computing using Cu0.1Te0.9/HfO2/Pt diffusive memristors. *Nature Communications* **13**, 1–8 (2022).

9. A. Alaghi, W. Qian, J. P. Hayes, The promise and challenge of stochastic computing. *IEEE Transactions on Computer-Aided Design of Integrated Circuits and Systems* **37**, 1515–1531 (2018).
