# Supplementary material for: Lightweight error-tolerant edge detection using memristor-enabled stochastic computing
Source: Nat Commun. 2025 May 16;16:4550. doi: 10.1038/s41467-025-59872-2 (PMC12084603; doi:10.1038/s41467-025-59872-2)
Supplement: Supplementary file 2 — Description of Additional Supplementary Files [file 41467_2025_59872_MOESM2_ESM.pdf]

Description of Additional Supplementary Files

Supplementary Movie 1: Large-scale edge detection simulation on *The Horse in Motion*. The input data are encoded into 256-bit stochastic numbers.

Supplementary Movie 2: Large-scale edge detection simulation on vehicles on highway for road scene parsing. The input data are encoded into 256-bit stochastic numbers.

Supplementary Movie 3: Large-scale edge detection simulation on real-time MRI for speech rehabilitation. The input data are encoded into 256-bit stochastic numbers.
